# Person-centered osteopathic practice: patients’ personality (body, mind, and soul) and health (ill-being and well-being)

**DOI:** 10.7717/peerj.1349

**Published:** 2015-10-27

**Authors:** Elin Fahlgren, Ali A. Nima, Trevor Archer, Danilo Garcia

**Affiliations:** 1 Network for Empowerment and Well-Being, Gothenburg, Sweden; 2 Dresden International University, Dresden, Germany; 3 Osteopathie Schule Deutschland, Hamburg, Germany; 4 Department of Psychology, University of Gothenburg, Gothenburg, Sweden; 5 Blekinge Center of Competence, Blekinge County Council, Karlskrona, Sweden; 6 Centre for Ethics, Law and Mental Health (CELAM), University of Gothenburg, Gothenburg, Sweden

**Keywords:** Character, Cloninger’s model, Personality, Patient-centered care, Person-centered care, Osteopathy, Biopsychosocial model, Temperament, Ternary Structures, Unity of being, Osteopathic medicine

## Abstract

**Background.** Osteopathic philosophy and practice are congruent with the biopsychosocial model, a patient-centered approach when treating disease, and the view of the person as a unity (i.e., body, mind, and soul). Nevertheless, a unity of being should involve a systematic person-centered understanding of the patient’s personality as a biopsychosociospiritual construct that influences health (i.e., well-being and ill-being). We suggest Cloninger’s personality model, comprising temperament (i.e., body) and character (i.e., mind and soul), as a genuine paradigm for implementation in osteopathic practice. As a first step, we investigated (1) the relationships between personality and health among osteopathic patients, (2) differences in personality between patients and a control group, and (3) differences in health within patients depending on the presenting problem and gender.

**Method.** 524 osteopathic patients in Sweden (age mean = 46.17, *SD* = 12.54, 388 females and 136 males) responded to an online survey comprising the Temperament and Character Inventory and measures of health (well-being: life satisfaction, positive affect, harmony in life, energy, and resilience; ill-being: negative affect, anxiety, depression, stress, and dysfunction and suffering associated to the presenting problem). We conducted two structural equation models to investigate the association personality-health; graphically compared the patients’ personality *T-scores* to those of the control group and compared the mean raw scores using *t-tests*; and conducted two multivariate analyses of variance, using age as covariate, to compare patients’ health in relation to their presenting problem and gender.

**Results.** The patients’ personality explained the variance of all of the well-being (*R*^2^ between .19 and .54) and four of the ill-being (*R*^2^ between .05 and .43) measures. Importantly, self-transcendence, the spiritual aspect of personality, was associated to high levels of positive emotions and resilience. Osteopathic patients, compared to controls, scored higher in six of the seven personality dimensions. These differences were, however, not considerably large (divergences in *T-scores* were <1 *SD*, *Cohen’s d* between 0.12 and 0.40). Presenting problem and gender did not have an effect on any of the health measures.

**Conclusion.** The patient’s personality as a ternary construct (i.e., body, mind, and soul), which is in line with osteopathy, is associated to both well-being and ill-being. The lack of substantial differences in personality between patients and controls implies that the patients had not any personality disorders. Hence, osteopaths might, with proper education, be able to coach their patients to self-awareness. The lack of differences in health variables between osteopathic patients with different presenting problems suggests that practitioners should focus on the person’s health regardless of the type of presenting problem.

“*You are not a drop in the ocean. You are the entire ocean in a drop*.”

رومی محمد الدینجلال [Jalāl ad-Dı¯n Muhammad Rūmı¯]

Osteopathy is a drug-free non-invasive manual therapy practice with a health-orientated, patient-centered, and holistic view of the human being (Thomson, Petty & Moore, 2013; Penney, 2013). For instance, the four basic tenets of osteopathic philosophy are: (1) the body is a unit, the person a unit of mind, body, spirit (or soul^1^1Although the term spirit is common in osteopathic philosophy, for the rest of this article we refer to this part of the human being as spirituality and/or soul. We see it as appropriate here, despite the fact that the term soul is not common in any other medical practice but perhaps some other type of alternative medicine. After all, the Greek word *Psyche* found in *psych*ology and *psych*iatry stands for “life, soul, or spirit,” which is distinct from *soma*, which refers to the “body” (Cloninger, 2004; see also Cloninger & Cloninger, 2011a; Cloninger & Cloninger, 2011b; Cloninger, Salloum & Mezzich, 2012).), (2) the body is capable of self regulation, healing and health maintenance, (3) structure and function are reciprocally interrelated, and (4) treatment ought to be based on the three tenets above (Seffinger et al., 2003). In other words, osteopathic practice focuses not only upon interrelationships between all parts and systems of the body for optimal function and health, but also on the person as a whole in relation to physical, psychological, social, and spiritual aspects of health and the being. Accordingly, health pertains to not merely the absence of disease or infirmity (i.e., ill-being), but also as a state of physical, mental and social well-being (World Health Organization, 1946). In this context, well-being incorporates the notions of feeling good (i.e., happiness), doing good (i.e., mature and actively virtuous living), enjoying physical health (i.e., absence of disease or infirmity), and prosperity (i.e., success, good fortune, and flourishing) (Cloninger, 2004; Cloninger, 2013).

In this framework, the philosophy of osteopathic practice is congruent with the biopsychosocial model (Penney, 2013)—a scientific model that refers to a dynamic and complex interaction of physiological, psychological, and social factors that can both result in and contribute to illness (Engel, 1980; Gatchel et al., 2007). As patient-centered practitioners, osteopaths aim to focus on helping the patient through an on-going practitioner-patient relationship (cf. Engel, 1980). Through this relationship, the osteopath gathers information and data in order to develop a hypothesis, presents a diagnosis and a treatment plan (cf. Engel, 1980). Data may be assembled both through psychological and behavioral means, such as, clinical and medical examinations, subjective reports, and the patient’s behavior and demographics (Engel, 1980). Nevertheless, the patient-centered approach is not fully reconcilable with the basic tenets of osteopathic philosophy (Paulus, 2013; Thomson, Petty & Moore, 2013). The term, patient, and the manner in which biopsychosocial patient data is gathered, for example, implies a reduction of the person to a passive recipient of healthcare (Thomson, Petty & Moore, 2013). In contrast, the four tenets of osteopathic philosophy suggest that treatment plans should be highly personalized to comprise the whole being (Seffinger et al., 2003) in order to empower the person to heal her/himself. Furthermore, the spiritual aspect of the ‘being’ is not explicitly included in the biopsychosocial model, which was constructed to consider the missing psychological, social, and behavioral dimensions of illness in the biomedical model (Engel, 1977; Engel, 1980; Gatchel et al., 2007; Penney, 2013). Certainly, today’s health care practitioners remain uncomfortable with the concept of spirituality (Paulus, 2013), possibly due to its perception as an abstract and non-scientific concept that, if it is not reliably measured, might lead to potential harm and other costs (Pergolizzi, 2015).

We propose that osteopathy might benefit from moving from a patient-centered approach to a person-centered one, in order for practitioners to understand the whole being and her/his health. The use of a person-centered approach in health care, in contrast to a patient-centered one, focuses in individual interrelationships over time, views diseases as interrelated phenomena, views body systems as interrelated, allows for the person’s own health concerns in their own treatment, and follows the development of people’s health problems as well as the diseases (see Starfield, 2011, for a clear differentiation between patient-centered and person-centered care). Moreover, a person-centered approach in health care puts emphasis on the need for the practitioner to understand who the person really is in order to address the therapeutic needs of the whole person and help the person to heal her/himself by learning to live well (Wong & Cloninger, 2010). That is, a person-centered practitioner helps the individual to become aware of behaviors that generate ill-being (e.g., stress, depression, anxiety, pain), but also to become aware of her/his ability to make self-directed choices that foster well-being (e.g., life satisfaction, positive emotions) (Cloninger, 2004; Wong & Cloninger, 2010; Cloninger, Salloum & Mezzich, 2012).

Importantly, if osteopaths aim to understand the person as a unity of being (i.e., body, mind, and soul) in the framework of a biopsychosocial model, then they need a scientifically robust paradigm that also includes a person’s spiritual values. Otherwise, they risk limiting or entrapping their own actions and those of the patients by creating unnecessary boundaries, such as, body-mind duality (Borell-Carrió, Suchman & Epstein, 2004). The lack of a scientific paradigm also risks that treatment is based on the osteopath’s own values or dogmas, thus, allowing the practitioner to freely emphasizing the ‘bio’, ‘psycho’, ‘social’, or ‘spiritual’ aspect without any rationale behind (Ghaemi, 2009). Fortunately, Cloninger’s biopsychosocial model of personality (see Cloninger, Svrakic & Przybeck, 1993; Cloninger, 2004) has over 20 years of scientific history behind. According to Cloninger’s model, personality development and health outcomes from this development is an on-going epigenetic procedure in which temperament (i.e., body) and character (i.e., mind and soul) generates behavior by interacting and influencing one another and adapting to external events (i.e., social) (Cloninger, Svrakic & Przybeck, 1993; Cloninger, 2013). Cloninger’s model is, for instance, the only personality model that comprises a ternary structure of personality (body, mind, and soul) by defining the spiritual or soul aspect of the being, self-transcendence, as one branch of the person’s mental self-government that allows her/him to be creative, intuitive, spiritual, and to appreciate beauty and have a sense of being part of something bigger that the self.^2^2For other models that have later addressed similar but incomplete aspects of a person’s spirituality (Cloninger, 2005), see the Virtues in Action Classification by Peterson & Seligman (2004).

Before discussing the importance of a systematic and genuine knowledge of the patient’s personality, we outline Cloninger’s biopsychosocial model of personality as the paradigm for the understanding of the person’s drives and emotions (i.e., body) but also her/his self-concept (i.e., who he/she is, what she/he is, and why she/he is here) in three dimensions related to the self, others, and something bigger than the self (e.g., God, nature, all humanity).

## Cloninger’s biopsychosocial model of personality: temperament and character dimensions

Personality may be defined as the “dynamic organization within the individual of the psychobiological systems by which the person both shapes and adapts uniquely to an ever-changing internal and external environment” (Cloninger, 2012). According to Cloninger, human personality has evolved through three major systems of learning and memory in a long series of steps through evolution (Cloninger, 2004; Cloninger, 2008). The first one is the procedural system, which regulates different emotional responses such as anger, fear, disgust, and ambition, that is, the temperament dimensions of personality. Temperament involves automatic responses to perceptual stimuli and is defined as individual differences in associative learning in four dimensions (Cloninger, 1987; Cloninger, Svrakic & Przybeck, 1993): *Novelty seeking* (1), associated with the neurotransmitter dopamine, is the tendency of frequent activation or initiation of behaviors in response to novel stimuli, potential rewards, and punishments expressed as frequent exploration of new unfamiliar places or situations, quick loss of temper, impulsive decision making, and active avoidance of monotony; *Harm avoidance* (2), associated with the neurotransmitter serotonin, is the tendency to avoid or cease behaviors due to intense response to aversive stimuli expressed as fear of uncertainty, shyness of strangers, quick fatigability, and pessimistic worry of future problems; *Reward dependence* (3), associated with the neurotransmitter noradrenaline, is the tendency to respond intensively to reward expressed as sentimentality, social attachment, and dependence of approval of others; and finally *Persistence* (4), which is the tendency to persevere despite fatigue or frustration, overachieving, and perfectionism (see Table 1 for a definition of high/low levels in each temperament dimension). In the context of a holistic view of the human being, the temperament domain and its dimensions represent the biological or body aspect of personality. Importantly, the temperament dimensions are useful to predict disorders and destructive behaviors (e.g., substance abuse is associated to high levels of novelty seeking), but not sufficient to predict who will develop a disorder or maladaptive behaviors (Cloninger, 2004)—for example, not all individuals who are high in novelty seeking develop substance abuse problems.

**Table 1:** Description of the temperament and character dimensions.

	Temperament and character descriptors		
		High scorers	Low scorers
Temperament	Harm avoidance	Worrying and pessimistic; fearful and doubtful; shy; fatigable.	Relaxed and optimistic; bold and confident; outgoing; vigorous.
	Novelty seeking	Exploratory and curious; impulsive; extravagant and enthusiastic; disorderly.	Indifferent; reflective; frugal and detached; orderly and regimented.
	Reward dependence	Sentimental and warm; dedicated and attached; dependent.	Practical and cold; withdrawn and detached; independent.
	Persistence	Industrious and diligent; hard-working; ambitious and overachiever; perseverant and perfectionist.	Inactive and indolent; gives up easily; modest and underachiever; quitting and pragmatist.
	Self-directedness	Mature and strong; responsible and reliable; purposeful; resourceful and effective; self-accepted; habits congruent with long term goals.	Immature and fragile; blaming and unreliable; purposeless; inert and ineffective; self-striving; habits incongruent with long term goals.
Character	Cooperativeness	Socially tolerant; empathic; helpful; compassionate and constructive; ethical and principled.	Socially intolerant; critical; unhelpful; revengeful and destructive; opportunistic.
	Self-transcendence	Wise and patient; creative and self-forgetful; united with the universe.	Impatient; unimaginative and self-conscious; pride and lack of humility.

**Notes.**

Reproduced with permission from CR Cloninger.

**Figure 1:**
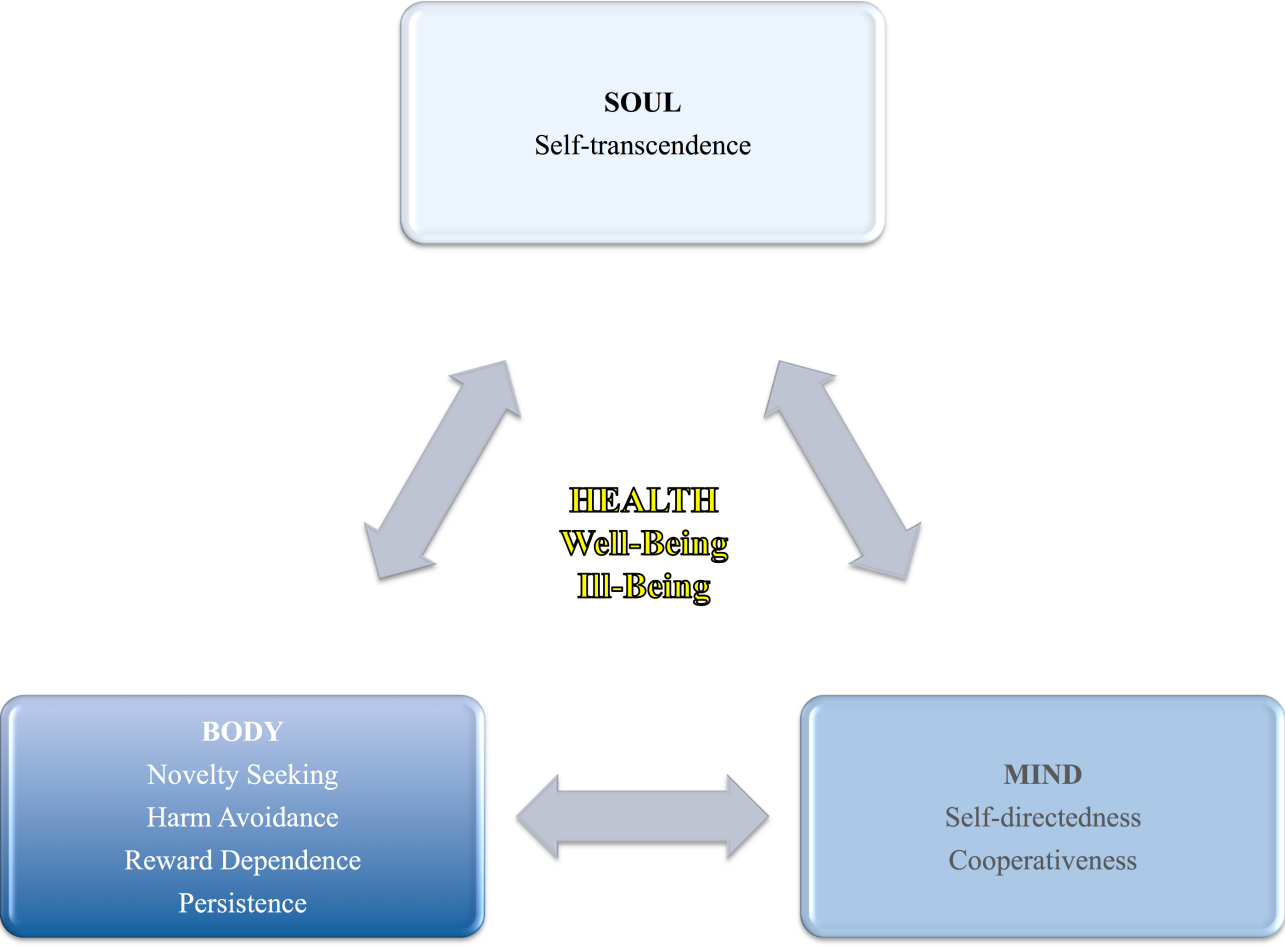
Health (i.e., well-being and ill-being) in relation to personality as a holistic view of the human being (i.e., body, mind, and soul).

The second system of learning and memory, the propositional system, is present in primates and helps the individual to be self-directed and cooperative in a social environment. The third system, the episodic system, exists only among humans and stands for humans’ capacity for self-awareness, which allows introspection and recollection of autobiographical memories (Cloninger, 2008). The second and third systems are responsible for the presence of three character dimensions, which can be defined as individual differences in values, goals and self-conscious emotions (e.g., hope, love, and faith) or what the individual makes of her/himself intentionally (Cloninger, Svrakic & Przybeck, 1993): *self-directedness* (1) refers to self-determination, being able to control, regulate, and adapt behavior in accordance to own goals and values, to be self-sufficient, self-acceptant, responsible, reliable, and effective; *cooperativeness* (2) accounts for individual differences in acceptance of and identification with other people, tolerance, helpfulness, and empathy; and *self-transcendence* (3) which refers to individual differences in selflessness or self-forgetfulness, patience, spirituality, and identification with something bigger than the self that gives meaning to one’s existence (Cloninger, Svrakic & Przybeck, 1993; Köse, 2003). See Table 1 for a definition of high/low levels in each character dimension. Hence, while the temperament dimensions represent the body aspect of personality; the character dimensions represent the psychological or mind aspect, but also the spiritual or soul aspect of personality (see Fig. 1).

Furthermore, prospective studies on the influence of parenting, during childhood, on adult personality, measured after 18 years, demonstrated that parenting and social norms influenced the development of character, but not temperament. It seems plausible that the key difference is that “procedural learning of habits and skills influences the conditioning of temperament, whereas propositional or semantic learning of goals and values influences the development of character” (Wong & Cloninger, 2010, pp. 203). According to this notion, while parenting can influence temperament, presumably by behavioral conditioning, an average effect on the general population does not always emerge. For example, in a 18-year prospective study in Finland in which parents of the participants (*N* = 1,083) had been directly observed in their homes during childhood and had answered questions about parenting attitudes, socioeconomic status, health behaviors, and role satisfaction; parental care-giving and home-environment were *more strongly* associated with the participants character traits (especially self-directedness and cooperativeness) than with their temperament traits (Josefsson et al., 2013b). Relatedly, changes in mean levels of character traits are much greater between 20 and 45 years of age than for temperament traits (Josefsson et al., 2013a). In sum, in contrast to temperament, character matures with age and is influenced by socio-cultural learning and familial environmental factors, such as parenting style and socio-cultural norms. With regard to the biopsychosocial model, Cloninger’s definition and assessment of personality does certainly involve, besides the person, also a dynamically interrelation with the social environment (i.e., the ‘social’ part of the biopsychosocial model) since Cloninger’s model acknowledges that the person both shapes and is shaped by her/his external environment.

Cloninger (Cloninger, Svrakic & Przybeck, 1993) developed the Temperament and Character Inventory to measure these seven personality dimensions. This instrument has been used widely in the investigation of personality’s neurobiological foundations, together with other research technologies, such as, molecular neuroimaging (Borg et al., 2003), structural neuroimaging (Yamasue et al., 2008), and genetics (Mousavi et al., 2015). In addition, the Temperament and Character Inventory has been translated into and validated in several languages, such as, Swedish (Brändström et al., 1998), Dutch (De la Rie, Duijsens & Cloninger, 1998), Japanese (Kijima, Tanaka & Suzuki, 2000), Turkish (Köse et al., 2002), Finnish (Josefsson et al., 2013a), and Spanish (Gutierrez & Torrens, 2001). These studies show stringent psychometric properties comparable to what was obtained for the original American English version. Furthermore, there is normative data in 22 countries (e.g., USA, Sweden, Japan, China, Mexico, Italy) on four continents (North and South America, Europe, Asia, Oceania) indicating that the factor structure is identical around the world. There is also data on age appropriate norms showing that the same dimensions are present across the lifespan as well (CR Cloninger, pers. comm., 2015). Hence, the Temperament and Character Inventory offers a paradigm that is appropriate for applications in osteopathic practice for personality assessment of the care-seekers.

## The patient’s personality and osteopathic practice

Individuals seeking osteopathic care or manual therapy present, most of the time, some kind of physical pain related to the musculoskeletal system (Penney, 2010). As osteopathy is a manual therapy practice, a large amount of osteopathic research relates to the body, its physiology, and treatment effects. Psychological outcomes are, however, also commonly investigated. For example, myofascial induction techniques (i.e., therapy that aims to relax contracted muscles, improve blood and lymphatic circulation, and stimulate the stretch reflex in muscles) decrease anxiety in adult healthy males (Fernández-Pérez et al., 2008). Massage increases positive affect and decreases both fidgeting behavior and anxiety among children, adolescents, and psychiatric patients with depression or dysthymic disorder (Field et al., 1992; see also Plotkin et al., 2001 for positive outcomes with regard to depression). Osteopathic treatment reduces also stress and has an effect on the autonomic nervous system. Henley and colleagues (2008), for instance, demonstrated that a cervical myofascial release technique affects the sympathovagal balance by shifting from the sympathetic nervous system to the parasympathetic nervous system within the autonomic nervous system. This intervention resulted in a decrease in heart rate and greater gland and intestinal activity (see also Korotkov et al., 2012; Saggio et al., 2011). Consequently, individuals change from a state of fight or flight to a state of calm that helps her/him to preserve energy. These findings suggest a clear connection between the treatment of the body and health outcomes above-and-beyond the mere alleviation of pain.

Treating pain may be a complex process since the scope of pain interacts with several dimensions or factors in a person’s life (Gatchel et al., 2007; Henschke, Kamper & Maher, 2015). For instance, maladaptive behavior or destructive coping strategies (e.g., lack of physical exercise, smoking and drinking), may alter bodily functions that can have a significant impact on structural integrity of the body, for example, obesity or inactiveness resulting in cardiovascular diseases or type II diabetes (Cooper, 2013). This type of maladaptive behavior is related to, besides genetic factors, the person’s inability to make self-directed choices with regard to her/his health (see, for example, Mokdad et al., 2004, who show that individual differences in personality, lifestyle, and stress account for large prevalence of mortality from physical disorders in the USA). Thus, although most of osteopathic research only focuses on the effect of body-related variables (e.g., the experience or alleviation of pain) on body and mind related outcomes (e.g., cortisol levels, self-reported stress levels), there are clear associations between the patients’ personality to her/his health.

There is evidence, for example, that high levels of harm avoidance and low levels of self-directedness are linked to nonspecific musculoskeletal pain (Malmgren-Olsson & Bergdahl, 2006), fibromyalgia (García-Fontanals et al., 2014) as well as chronic pain (Conrad et al., 2013; Conrad et al., 2007). A person who is high in harm avoidance and low in self-directedness presents a patient profile that is identified by cautiousness, pessimism, insecurity, and with difficulties accepting responsibilities, expressing chronically low self-esteem, lacking long-term goals, and conflict of identity (Cloninger, 2004; Malmgren-Olsson & Bergdahl, 2006). High levels of harm avoidance together with low levels of self-directedness are linked therefore also to an individual’s tendency to have a heightened perception of pain, pain related anxiety, fear-avoidance, avoidant coping strategies, misinterpretation of signals, depression, anxiety, and catastrophic thinking (Cole, 2002; Conrad et al., 2013; Eccleston, 2001; Knaster et al., 2012; Linton, 2000; Pud et al., 2004; Vranceanu et al., 2014). In short, this personality profile has a significant role in different aspects of pain (e.g., its etiology, response to, intensity, duration and level of disability). Pain itself may in turn influence a person’s way of interacting with others and her/his daily activities (Keefe et al., 2004). In this context, character traits, which can modify and change the automatic behaviors and emotions derived from temperament traits (Cloninger, 2004), are positively associated to psychophysiological coherence, a state of calm alertness that occurs naturally with sustained positive emotions. Slow, deep breathing, relaxing, and sleeping can induce psychophysiological coherence; which in turn, increases efferent parasympathetic activity (Zohar, Cloninger & McCraty, 2013). This suggests at least phenotypical, if not causal, relations among personality, heart rate variability, and health.

We argue that, in order to help the patient become aware of their behavior and be able to modify it (cf. Penney, 2013), a genuine integration of the biopsychosocial model and the concept of unity of being in osteopathic practice are in need of Cloninger’s personality model as the valid paradigm. This will allow practitioners to understand the drives and motives behind a person’s behaviors and their relationship to both well-being and ill-being. Hence, a systematic and intuitive knowledge about the person in front, her/his temperament and character, is a valuable tool for the practitioner to help that person to increase her/his self-awareness and ability to differentiate between healthy and unhealthy behaviors (Wong & Cloninger, 2010).

## The present study

The biopsychosocial model of personality developed by Cloninger offers a valid paradigm for osteopathic practice that might lead to improvement and change in response to treatment by enabling better treatment planning and estimation of disease progression (Conrad et al., 2013). Research regarding pain using Cloninger’s model has been applied generally within the scope of chronic pain (Conrad et al., 2013; Conrad et al., 2007; Weisberg & Boatwright, 2007) or in relation to psychotherapeutic or pharmaceutical treatment (Conrad et al., 2007). The aim of the osteopathic treatment is, through manual hands-on diagnosis and treatment, to enhance the patient’s ability and capacity to adapt, react, and cope to her/his environment, as well as to restore and maintain optimal function and health (Kuchera, 2007; Seffinger et al., 2003). It is not yet clearly understood how this is achieved in practice. An increased systematic and intuitive knowledge of the patients’ personality would move osteopathic practice beyond the limits of physical treatment and patient-centered care to a person-centered care in which the osteopaths may be able to help the patient to restore health by empowering the person to become more self-aware of her/his daily choices (cf. Eccleston, 2001; Linton & Shaw, 2011; Thomson, Petty & Moore, 2013). For example, to be able to recognize stress reactions and different coping strategies as patterns of personality would provide practitioners with information for prevention and early treatment or handling of many musculoskeletal disorders (Ahlberg et al., 2002) as well as to promote health and well-being and behaviors that help the individual to take care of her/his whole being (i.e., body, mind, and soul).

In addition to the necessity of a ternary model of personality (i.e., body, mind, and soul), we argue further that patients need to be seen as persons in the context of not only ill-being but also well-being. Osteopathic practitioners meet patients with a wide variety of presenting problems, pains and ailments, thus, a broad perspective of the osteopathic patient covering aspects of personality, and health as both well-being and ill-being would be beneficial for osteopathy as a health-orientated therapy and practice (Penney, 2013). As a primary step along this path, the aims of the present study were:

1. to investigate the relationships between personality dimensions, well-being, and ill-being among osteopathic patients.
2. to investigate if osteopathic patients differ in personality compared to non-patients in a control group.
3. to investigate if there were any differences in ill-being and well-being between osteopathic patients, depending on their presenting problem.

We expected personality variables to predict the patients’ health as shown in earlier studies. For example, low harm avoidance and high self-directedness were expected to be associated to high levels of well-being and low levels of ill-being (see, among others, Cloninger & Zohar, 2011). Both self-directedness and cooperativeness are markers of mental health in western cultures (Cloninger, 2004), thus, cooperativeness was also expected to be associated to the patient’s health as well. The relationship between patients’ self-transcendence to their health was of special interest because self-transcendence represents the spiritual or soul aspect of personality. An aspect of the being that is important in osteopathic philosophy. Accordingly, well-being measures such as positive emotions are highest only when all three character dimensions are also high (Cloninger, 2004). If so, Cloninger’s model may offer an interpretation of osteopathic patients’ health in the context of the being as a unity of body, mind, and soul. In addition, investigating differences in personality between patients and healthy controls is important. After all, osteopathic practice in Sweden does not involve treatment of personality or psychiatric disorders. If osteopaths would be able to facilitate individuals’ character development thereby improving health, it is important that their patients are not in need of professional psychiatric care.

## Method

### Ethical statement

The study has ethical approval from the Osteopathic Research Institute’s ethics commission (Ethik-Kommission des Osteopathic Research Institute, Hamburg, Germany). The participants are protected by informed consent process, they are all above the age of 18, and were informed of what is being collected and were repeatedly given the option to withdraw their consent and cease their participation.

### Participants and procedure

**Figure 2:**
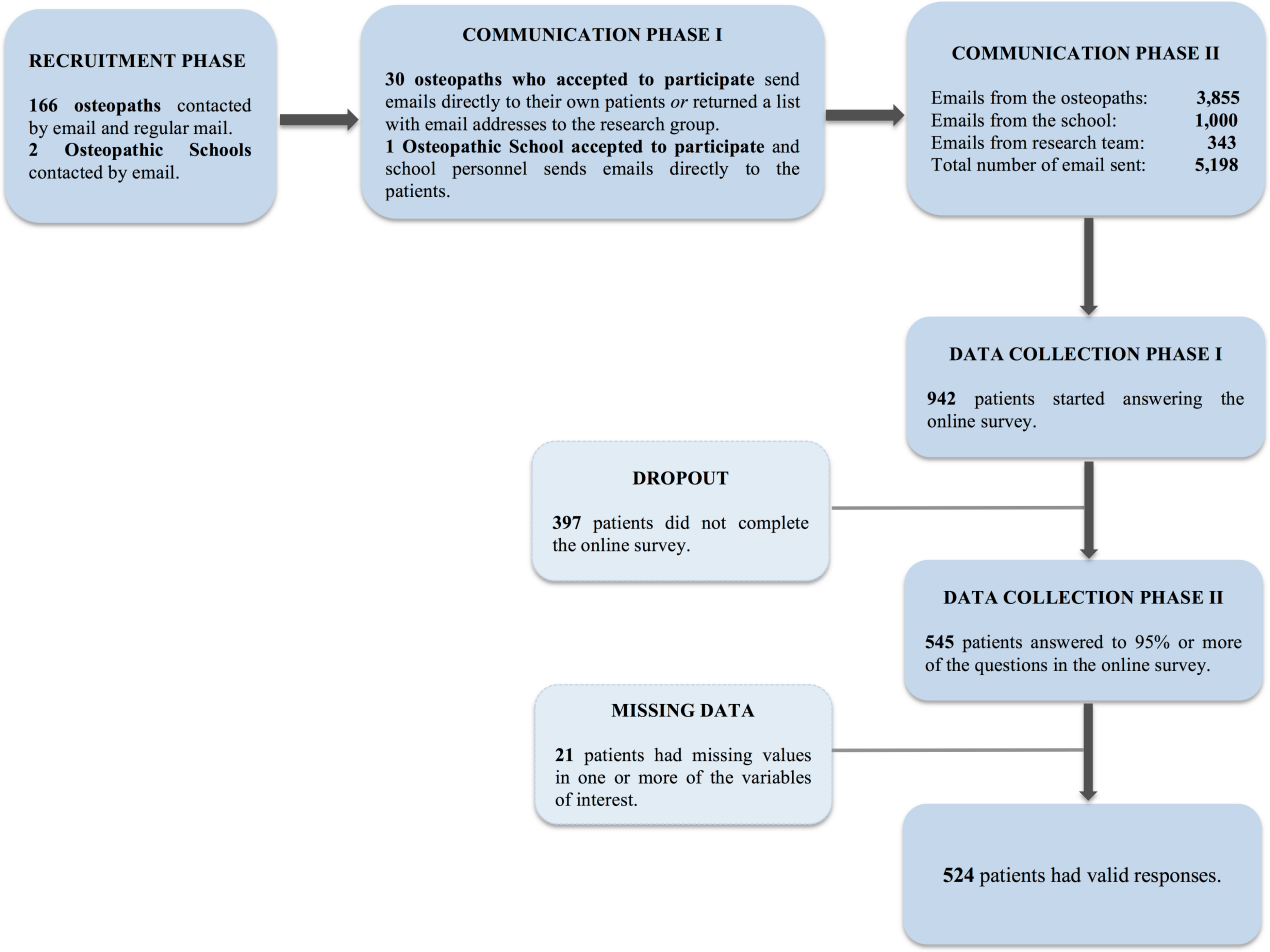
Flow chart over the participant recruitment procedure.

Participants were contacted through 166 osteopaths who are members of the Swedish Osteopathic Association (see Fig. 2 for a flow chart of the participant recruitment procedure). The contact with the osteopaths was conducted through email and regular mail delivery. The 30 osteopaths who agreed to help with the data gathering were asked to, on voluntary basis, email or administer a link with the questionnaires to their patients in treatment, and report back how many patients they had emailed or contacted. For those who did not have the email addresses of their patients or found it more convenient, they had, with the approval of their patients, the possibility to gather patients’ email addresses on a list that was included in the regular mail delivery as well as an envelope for return. Osteopaths could then send the list to one of the members of the research team who provided the link to the patients via email. To motivate the osteopaths to help with the gathering of the data, they were offered a cinema ticket if they emailed, or gathered, at least 15 email addresses. The two existing clinics at the osteopathic schools in Sweden were also contacted for participation and administration of the link with the questionnaire to their patients; only one school was willing to participate. The email sent to osteopaths and the school clinic contained information about the study and text that the osteopaths and administration staff at the school could copy and paste and then email to their patients. This text contained information about the study and that upon participation each person would enter a lottery to win one cinema ticket out of 250 tickets. The first page of the questionnaire provided information regarding handling of data and anonymity. The very last question allowed subjects to agree or not to be contacted for voluntary participation in further studies.

The known total of patients were 5,198 whom received an email with the link to the questionnaires (3,855 were sent directly by osteopaths to patients, 1,000 from the school to patients in the school clinic and 343 were sent from the research team). A total of 942 patients started the survey and 545 completed 95% or more of the questions (valid response rate = 68%). See Table 2 for demographics. The analyses were at first conducted with this last number of participants and due to missing values in all variables of interest, the final number of valid responses was 524 (valid response rate: 65%). This sample had an age mean = 46.17 (std. = 12.54) and comprised of 388 females and 136 males.

**Table 2:** Demographics and medicine intake among patients in the present study.

**Age** (*n* = **524**)	
Mean ± SD	46.17 ± 12.54
**Gender**	
Female	404 (74.1%)
Male	141 (25.9%)
**Total**	545 (100%)
**Education (years after primary school)**	
0	16 (2.9%)
1–3	173 (31.7%)
4–7	248 (45.5%)
>7	105 (19.3%)
**Total**	542 (100%)
**Occupation**	
Working	439 (80.6%)
Unemployed	11 (2.0%)
Student	29 (5.3%)
Sick leave	15 (2.8%)
Parental leave	19 (3.5%)
Retired	23 (4.2%)
**Total**	536 (100%)
**Location of pain**	
Lower back	150 (27.5%)
Upper (mid) back	38 (7.0 %)
Neck and/or shoulder	64 (11.7%)
Hip/knee/feet	15 (2.8%)
Other	67 (12.3%)
Two or more of above	211 (38.7%)
**Total**	545 (100%)
**Usage of painkillers**	
Never	201 (36.9%)
Once/month	207 (38.0%)
Once/week	68 (12.5%)
2–3 times/week	39 (7.2%)
All the time	29 (5.3%)
**Total**	544 (100%)
**Usage of psychotrophics**	
Never	495 (90.8%)
Once/month	7 (1.3%)
Once/week	2 (0.4%)
All the time	39 (7.2%)
**Total**	543 (100%)

#### Measures

##### Personality

The Temperament and Character Inventory—Revised (Cloninger, Svrakic & Przybeck, 1993) assess the four temperament and three character dimensions using 140 items and a five-point Likert scale (1 = *definitely false*, 5 = *definitely true*). Examples of questions from the four temperament dimensions are: harm avoidance, “I often feel tense and worried in unfamiliar situations, even when others feel there is little to worry about” (*Cronbach’s alpha* = .86); novelty seeking, “I often try new things just for fun or thrills, even if most people think it is a waste of time” (*Cronbach’s alpha* = .74); reward dependence, “I like to discuss my experiences and feelings openly with friends instead of keeping them to myself” (*Cronbach’s alpha* = .71); and persistence, “I often push myself to the point of exhaustion or try to do more than I really can” (*Cronbach’s alpha* = .86). Examples of questions from three character dimensions: self-directedness, “In most situations my natural responses are based on good habits that I have developed” (*Cronbach’s alpha* = .88); cooperativeness, “I often consider another person’s feelings as much as my own” (*Cronbach’s alpha* = .74); and self-transcendence, “I sometimes feel so connected to nature that everything seems to be part of one living organism” (*Cronbach’s alpha* = .89).

##### Well-being and ill-being measures

The Harmony in Life scale (Kjell et al., 2013; Kjell et al., 2015) assesses a global sense of harmony in one’s life (well-being). It consists of five statements (e.g., “My lifestyle allows me to be in harmony”) where participants are asked to indicate degree of agreement on a 7-point Likert scale (1 = *strongly disagree*, 7 = *strongly agree*). *Cronbach’s alpha* in the present study was .89.

The Satisfaction With Life Scale (Diener et al., 1985) measures global cognitive judgments of satisfaction with one’s life (well-being) using five items (e.g., “In most ways my life is close to my ideal”) and a seven-point Likert scale (1 = *strongly disagree*, 7 = *strongly agree*). This scale showed a *Cronbach’s alpha* = .89 in the present study.

The Connor–Davidson Resilience Scale modified version (Dong et al., 2013) is a self-rating scale that measures resilience and was created to use in clinical practice. The 27-items (e.g., “I am able to adapt to change”) use a five-point Likert scale (0 = *totally disagree*, 4 = *totally agree*) to measure well-being as the person’s ability to overcome adversity and to return to her/his previously established functional baseline. The reliability in the present study was *Cronbach’s alpha* = .92.

The Positive Affect Negative Affect Schedule (Watson, Clark & Tellegen, 1988) was used to measure positive affect (well-being) and negative affect (ill-being). Participants are asked to estimate and rate to which extent they have felt ten positive (e.g., interested, enthusiastic, proud) and ten negative (e.g., distressed, upset, guilty) feelings and moods during the last week on a five-point scale (1 = *very slightly or not at at all*, 5 = *extremely*). The instrument showed the following *Cronbach’s alphas* in the present study: positive affect = .91, negative affect = .86.

The Stress–Energy questionnaire (Kjellberg & Iwanowski, 1989) assesses experienced stress (ill-being) and energy (well-being) using 12 items and a six-point Likert scale (0 = *not at all*, 5 = *very much*). Examples of items for the stress scale are: tense, stressed, pressured (*Cronbach’s alpha* in the present study = .67). Examples of items for the energy scale are: active, energetic, focused (*Cronbach’s alpha* in the present study = .67).

The Hospital Anxiety and Depression scale (Zigmond & Snaith, 1983) consists of 14 items to measure ill-being using a four-point Likert scale (0 = *most of the time*, 3 = *not at all*), where seven items measure anxiety (e.g., “I feel tense and ‘wound up’”) and the other seven measure depression (e.g., “I have lost interest in my appearance”). *Cronbach’s alphas* in the present study were: anxiety = .81, depression = .72.

##### Pain related measures

Presenting problem. Participants were also asked to report the problem they seek treatment for. Six choices were available for the participants: lower back pain with or without Ischialgia (1), upper (mid) back pain (2), neck and/or shoulder pain (3), hip, knee and/or feet pain (4), other (5), and two or more of the above (6). We constructed three groups based on these choices: lower back problem group (choice 1; *n* = 141), one problem group (choices 2–5; *n* = 179), and two or more problems group (choice 6; *n* = 204). The resulting frequencies indicated that the most prevalent problems were related to the lower back or patients having more than one problem, which is in line with recent research (e.g., Manchikanti et al., 2009; Penney, 2010).

Dysfunction and suffering from the presenting problem. Participants were asked to answer two questions related to the presenting problem for which they seek manual treatment: How would you grade the level of your problem/pain? How much does it prevent you in your daily life or influences your daily tasks? Both questions were answered in a ten-point Likert scale (1 = *nothing at all*, 10 = *extremely much*) and were highly positive correlated (*r* = .72, *p* < .001). The answers to both questions were summarized to form a dysfunction and suffering composite (ill-being).

#### Analyses and statistical treatment

For the investigation of the relationship between personality (temperament and character) and health (well-being and ill-being) we conducted two structural equation models using personality as the independent variable in both models and well-being, respectively, ill-being as the dependent variables in the first and second model. This analysis allowed us to control for the relationships between all variables in the models.

For the comparison of osteopathic patients’ personality to that of the control group, we firstly transformed the patients’ raw score in each personality dimension (i.e., to *T-scores*) using the means and standard deviations from 1,230 individuals between the ages of 18–71 (580 males, 680 females) from the Swedish general population (novelty seeking: 58.51 ± 8.73, harm avoidance: 52.09 ± 10.91, reward dependence: 66.61 ± 9.15, persistence: 65.23 ± 9.85, self-directedness: 75.57 ± 11.26, cooperativeness: 76.70 ± 9.14, and self-transcendence: 37.93 ± 9.92; S Rosza, pers. comm., 2015). This control group data is not published and was collected by an independent research team for the validation of the large version of the Temperament and Character Inventory—Revised. In short, a *T-score* of 50 represents the mean in the control group and 10 points corresponds to one standard deviation. In other words, a deviation of 10 above or below 50 indicates a divergence of one standard deviation from the mean score in relation to the control group. For instance, a temperament score >2 standard deviations *above* the mean indicates that the individual has an extreme temperament, while a character score >2 standard deviations *below* the mean indicates that the individual has an immature character (cf. Cloninger, 2004; Garcia, Anckarsäter & Lundström, 2013). In addition to this graphical/visual analysis of the *T-scores*, we also conducted *t-tests* to identify significant differences between participants’ mean raw scores and the means from the control group.

For the analysis of potential differences in health (well-being and ill-being) between patients with different presenting problems we conducted two Multivariate Analysis of Covariance. Here we used the presenting problem groups (lower back problem, one problem group, and two or more problems) and gender as the factors in both analyses and well-being, respectively, ill-being as the dependent factors in the first and second analyses. In this context it is worth mentioning that lower back pain is the most common pain condition across the world (Hoy et al., 2012; Manchikanti et al., 2009), and the prevalence of lower back pain, neck pain, and chronic pain, for instance, is higher in women and increases with age (Hoy et al., 2012; Fejer, Kyvik & Hartvigsen, 2006; Manchikanti et al., 2009; Helme & Gibson, 1999). Hence, age was included as a covariate in both analyses and a three-group solution was found plausible to get almost equally large groups of presenting problem categories. Nevertheless, preliminary analyses were conducted using all presenting problem groups: (1), upper (mid) back pain (2), neck and/or shoulder pain (3), hip, knee and/or feet pain (4), other (5), and two or more of the above (6). This six-group categorization of the presenting problems did not have an effect on either well-being (*F*(25, 1762) = .98; *p* = .49, *Partial η2* = .01, *Wilks’ Lambda* = .95) or ill-being (*F*(25, 1688) = .97; *p* = .50, *Partial η2* = .01, *Wilks’ Lambda* = .95).

##### Missing data

*Little’s Chi-Square* test for Missing Completely at Random showed a *χ*^2^ = 163.29 (*df* = 179, *p* = .79) for women in the lower back problem group, a *χ*^2^ = 212.29 (*df* = 214, *p* = .52) for women in the one problem group, a *χ*^2^ = 106.69 (*df* = 126, *p* = .89) for women in the two or more problems group, a *χ*^2^ = 178.14 (*df* = 142, *p* = .02) for men in the lower back problem group, *χ*^2^ = 113.82 (*df* = 111, *p* = .41) for men in the one problem group, and a *χ*^2^ = 762.56 (*df* = 80, *p* = .59) for men in the two or more problems group. Thus, the Expectation–Maximization Algorithm was found suitable to use to input missing values.

##### Univariate outliers

To reduce the impact of variables with outliers we first standardized the scores of each variable and tested if any cases had larger standardized scores than ±3.29, as recommended by Tabachnick & Fidell (2007). The analysis did not detect any cases as univariate outliers.

##### Normality and linearity

We examined the distributions of the variables using scatter plots. Results show that variables have reasonably balanced distributions. The skewness for all varibles varied from 1.09 to 0.07 and kurtosis from 1.01 to .09. Because our sample size was relatively large (*N* = 545), these values of the skewness and kurtosis were judged as reasonable (see Wuensch, 2005; Tabachnick & Fidell, 2007).

##### Linearity and homoscedasticity

We also tested the predictors (i.e., temperament and character dimensions) in a standard multiple regression to produce the scatter plots of residuals against the dependent variables (i.e., the well-being and ill-being dimensions) to further examine linearity, and homoscedasticity (see Tabachnick & Fidell, 2007). The result indicated that the variables had reasonably balanced distributions and that there were no threats to linearity or homoscedasticity. Thus, the assumptions were met to conduct structural equation modeling.

##### Multivariate outliers

This analysis showed that, with 17 variables and a criterion *α* = .001, critical *x*^2^ = 40.79, that 21 values were considered as multivariate outliers and therefore removed. This procedure left a total of 524 participants that were used in all analyses presented here.

##### Multicollinearity and singularity

All the significant correlations among the variables in the present study were all below .69. This is below the recommended value of .90 or above (Tabachnick & Fidell, 2007, p. 88). In other words, there was no cause for worry about multicollianiarity and singularity in the present study.

##### Homogeneity of regression

The relationships between the dependent factors in the Multivariate Analyses of Covariance (i.e., well-being and ill-being variables) and the covariate (i.e., age) are similar for each group of each independent factor (i.e., gender and presenting problem). This means that the data had homogeneity of regression because all interactions between groups and age on the dependent variables are not significant. For the well-being analysis: age and gender (*F*(5, 508) = 1.29; *p* = .27, *Partial η2* = .01, *Wilks’ Lambda* = .99), age and presenting problem (*F*(10, 1016) = 1.31; *p* = .22, *Partial η2* = .01, *Wilks’ Lambda* = .98), and age, gender and presenting problem (*F*(10, 1016) = .88; *p* = .55, *Partial η2* = .01, *Wilks’ Lambda* = .98). For the ill-being analysis: age and gender (*F*(5, 508) = 1.14; *p* = .34, *Partial η2* = .01, *Wilks’ Lambda* = .99), age and presenting problem (*F*(10, 1016) = .53; *p* = .87, *Partial η2* = .01, *Wilks’ Lambda* = .99), and age, gender, and presenting problem (*F*(10, 1016) = .91; *p* = .53, *Partial η2* = .01, *Wilks’ Lambda* = .98). In other words, the assumptions were met to conduct the two Multivariate Analysis of Covariance.

##### Homogeneity of variance–covariance matrices

The *Box’s M* tests were not significant in any of the two Multivariate Analysis of Covariance (for the well-being analysis *p* = .09 and for the ill-being analysis *p* = .27) so, there was no threat against homogeneity of variance–covariance. In other words, the assumptions were met to conduct the two Multivariate Analysis of Covariance.

## Results

### Personality and well-being

**Figure 3:**
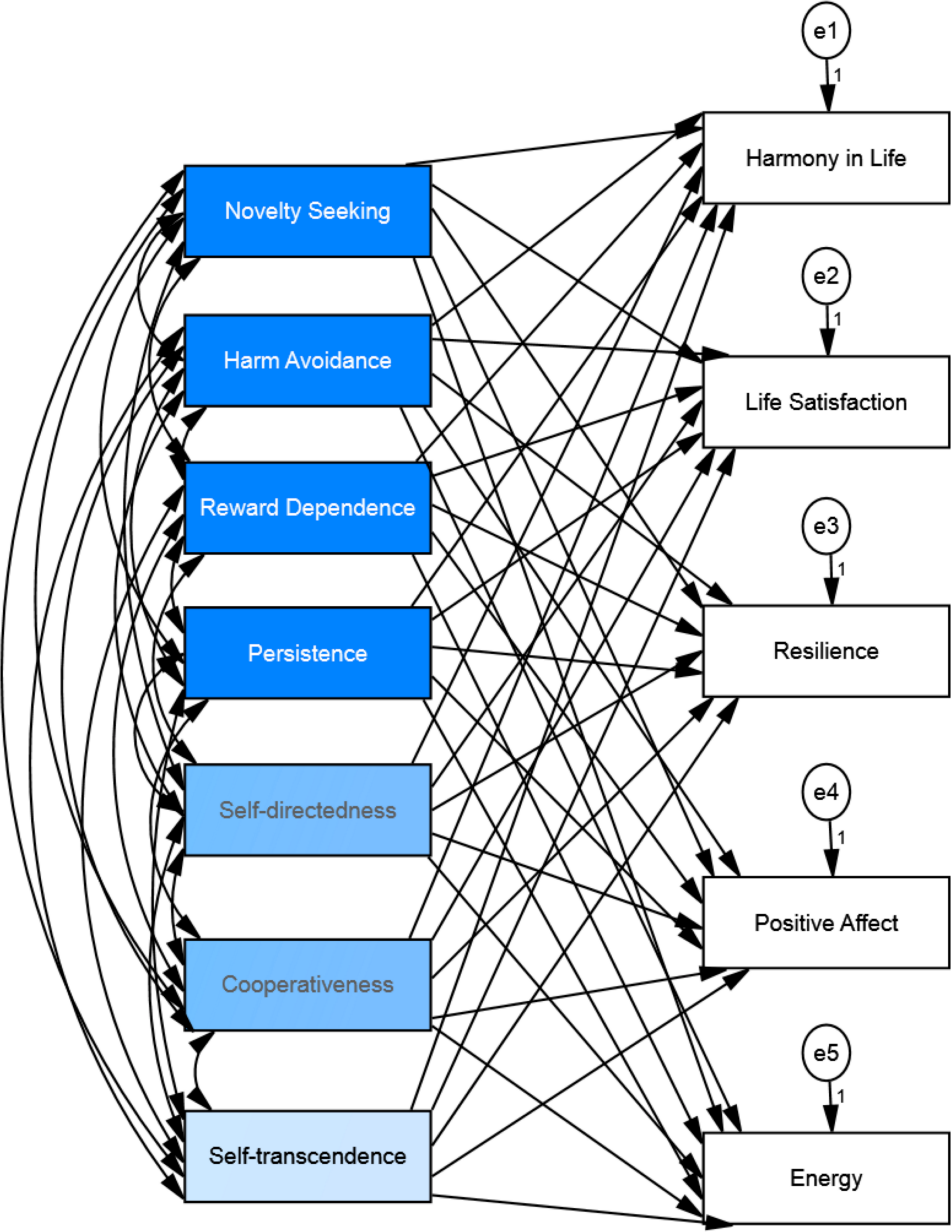
Hypothesized structural equation model of the relationship between personality and well-being among osteopathic patients.

**Figure 4:**
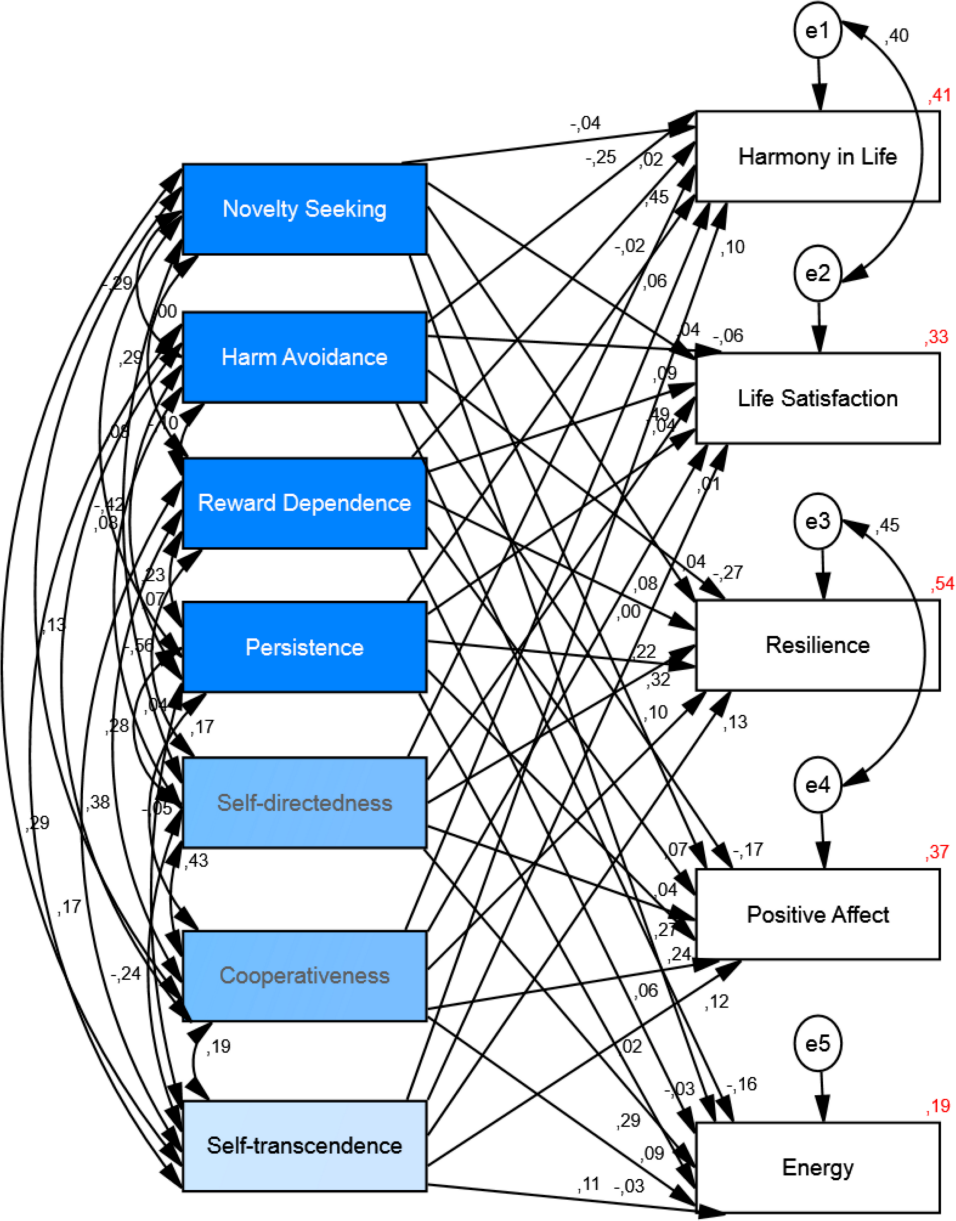
Structural equation model of the relationship between personality and well-being. The model shows the correlations among personality variables and the paths from personality to well-being and their standardized parameter estimates. *Chi-square* = 140.04, *df* = 8, *p* < .001; *goodness of fit index* = .96; *incremental fit index* = .94; *comparative fit index* = .94 and *normed fit index* = .94, (*N* = 524).

The structural equation model analysis for personality as the independent variable and well-being as the dependent variable (see Fig. 3) showed that *chi-square* value was significant (*Chi*^2^ = 350.73, *df* = 10, *p* < .001), the *goodness of fit index* was .88, the *comparative fit index* was .84, the *incremental fit index* was .85, and the *normed fit index* was .85. Thus, the *fit index* indicates that the model is not a good-fitting model. We added two correlation paths between residuals/errors (between harmony in life-life satisfaction and between resilience-positive affect) to obtain better fit of the model. The addition of these correlated residuals represents correlations that are explained outside of our model (see Fig. 4). The chi-square value for this new default model was smaller but still significant (*Chi*^2^ = 140.04, *df* = 8, *p* < .001). The chi-square statistic is influenced by sample size, so with larger samples does this lead to both larger value of chi-square statistic and likelihood of being significant (Kline, 2010). The *goodness of fit index* for the default model was .96, the *comparative fit index* was .94, the *incremental fit index* was .94 and the *normed fit index* was .94. Thus, indicating that the model, after the modifications, was a good-fitting model. The results of the structural equation model showed that personality could explain the variance of all well-being measures: harmony in life scale (*R*^2^ = .41), life satisfaction (*R*^2^ = .33), resilience (*R*^2^ = .54), positive affect (*R*^2^ = .37) and energy (*R*^2^ = .19). See Fig. 4 and Table 3 for the details.

**Table 3:** Standardized and unstandardized coefficients for the structural equation model of personality and well-being.

Predictor	Outcome	*β*	*B*	*Se*	*P*
Novelty seeking	Harmony in life *R*^2^ = .42	−.04	.00	.00	.30
**Harm avoidance**		−**.25**	−**.02**	**.00**	**.001**
Reward dependence		.02	.00	.00	.68
Persistence		−.02	.00	.00	.55
**Self-directedness**		**.45**	**.04**	**.00**	**.001**
Cooperativeness		.06	.01	.01	.15
**Self-transcendence**		**.10**	**.01**	**.00**	**.01**
Novelty seeking	Life satisfaction *R*^2^ = .33	.04	.01	.01	.28
Harm avoidance		−.06	−.01	.00	.24
**Reward dependence**		**.09**	**.01**	**.01**	**.03**
Persistence		.04	.00	.00	.36
**Self-directedness**		**.49**	**.05**	**.01**	**.001**
Cooperativeness		.01	.00	.01	.79
Self-transcendence		.00	.00	.00	.97
Novelty seeking	Resilience *R*^2^ = .54	.04	.00	.00	.21
**Harm avoidance**		−**.27**	−**.01**	**.00**	**.001**
**Reward dependence**		**.08**	**.00**	**.00**	**.01**
**Persistence**		**.32**	**.02**	**.00**	**.001**
**Self-directedness**		**.22**	**.01**	**.00**	**.001**
**Cooperativeness**		**.10**	**.01**	**.00**	**.001**
**Self-transcendence**		**.13**	**.01**	**.00**	**.001**
Novelty seeking	Positive affect *R*^2^ = .37	.07	.01	.00	.07
**Harm avoidance**		−**.17**	−**.01**	**.00**	**.001**
Reward dependence		.04	.00	.00	.27
**Persistence**		**.24**	**.02**	**.00**	**.001**
**Self-directedness**		**.27**	**.02**	**.00**	**.001**
Cooperativeness		.06	.00	.00	.17
**Self-transcendence**		**.12**	**.01**	**.00**	**.001**
Novelty seeking	Energy *R*^2^ = .19	−.03	.00	.00	.54
**Harm avoidance**		−**.16**	−**.01**	**.00**	**.001**
Reward dependence		.02	.00	.00	.68
**Persistence**		**.09**	**.01**	**.00**	**.03**
**Self-directedness**		**.29**	**.02**	**.00**	**.001**
Cooperativeness		−.03	.00	.01	.53
**Self-transcendence**		**.11**	**.01**	**.00**	**.01**

**Notes.**

Significant effects in bold type.

### Personality and ill-being

**Figure 5:**
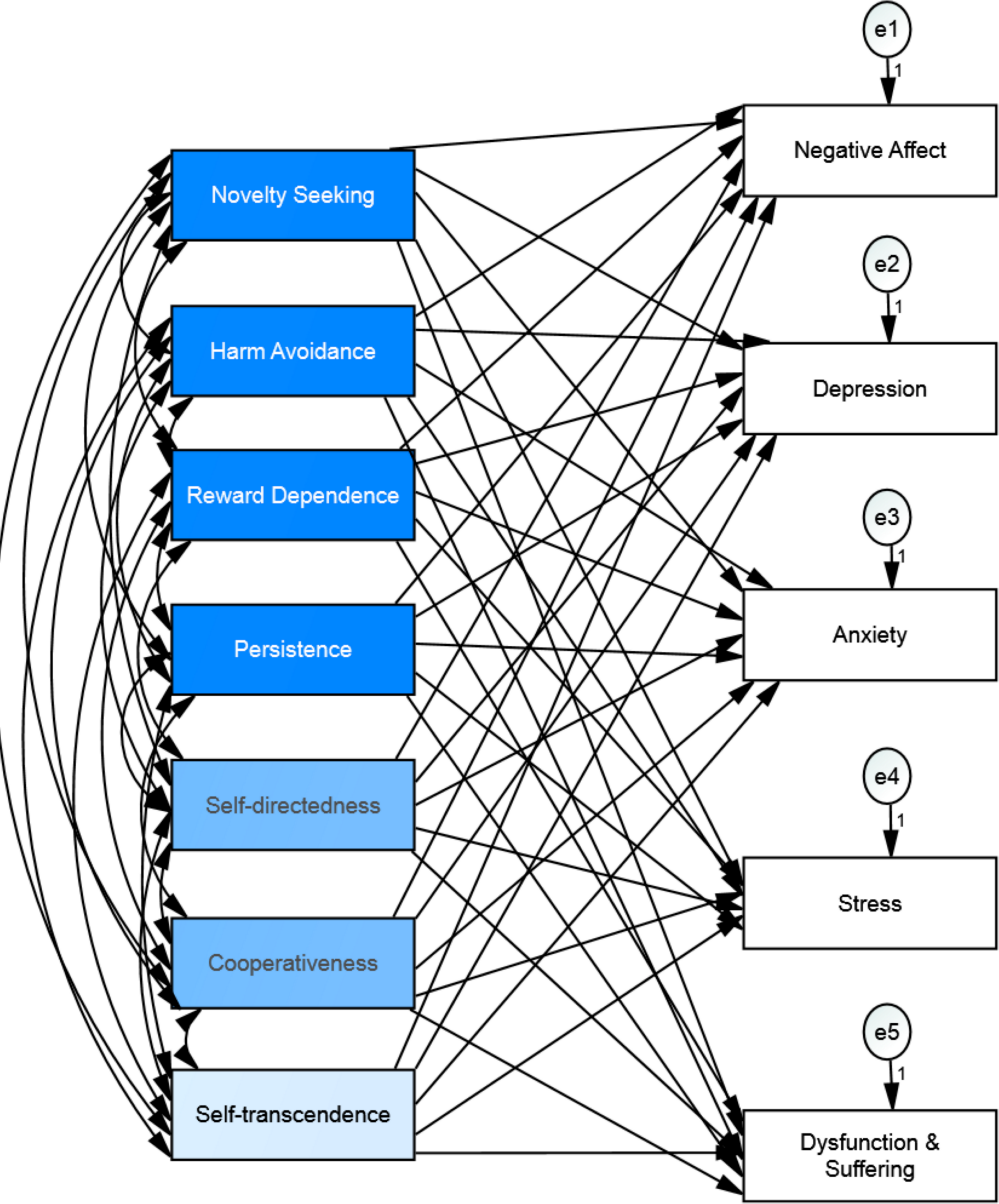
Hypothesized structural equation model of the relationship between personality and well-being among osteopathic patients.

**Figure 6:**
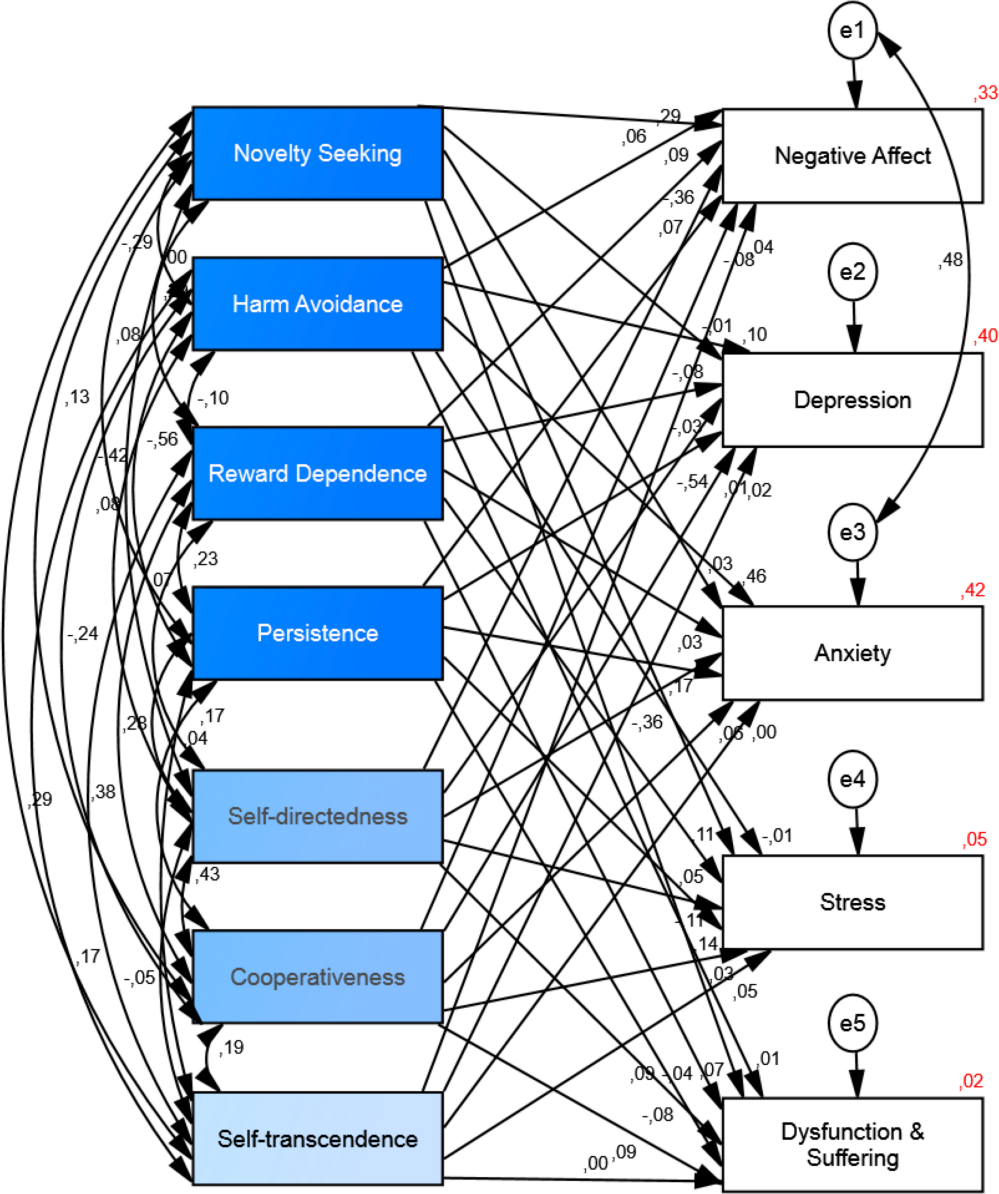
Structural equation model of the relationship between personality and ill-being. The model shows the correlations among personality variables and the paths from personality to ill-being and their standardized parameter estimates. *Chi-square* = 77.94, *df* = 9, *p* < .001; *goodness of fit index* = .97; *incremental fit index* = .96; *comparative fit index* = .96 and *normed fit index* = .96, (*N* = 524).

The structural equation model analysis for personality as the independent variable and ill-being as the dependent variable (se Fig. 5) showed that *chi-square* value was significant (*Chi*^2^ = 215.48, *df* = 10, *p* < .001), the *goodness of fit index* was .93, the *comparative fit index* was .87, the *incremental fit index* was .88 and the *normed fit index* was .87. Thus, the *fit index* indicates that the model is not a good-fitting model. Although the *goodness of fit index* (.93) indicated a good-fitting we added one correlation path between residuals/errors (negative affect-anxiety) to obtain a better fit of the model (see Fig. 6). After this modification the *chi-square* value for this new default model was smaller but still significant (*Chi*^2^ = 77.94, *df* = 9, *p* < .001). Again, the chi-square statistic is influenced by sample size so with larger samples does this lead to both larger value of chi square statistic and likelihood of being significant (Kline, 2010). The *goodness of fit index* for the default model was .97, the *comparative fit index was* .96, the *incremental fit index* was .96 and the *normed fit index* was .95. Thus, indicating that the model, after this modification, was a good-fitting model. The results of the structural equation model showed that personality could explain the variance of negative affect (*R*^2^ = .33), depression (*R*^2^ = .40), anxiety (*R*^2^ = .42), and stress (*R*^2^ = .05). See Fig. 6 and Table 4 for the details.

**Table 4:** Standardized and unstandardized coefficients for the structural equation model of personality and ill-being.

Predictor	Outcome	*β*	*B*	*Se*	*P*
Novelty seeking	Negative Affect *R*^2^ = .33	.06	.00	.00	.13
**Harm avoidance**		**.29**	**.02**	**.00**	**.001**
**Reward dependence**		**.09**	**.01**	**.00**	**.02**
Persistence		.07	.00	.00	.10
**Self-directedness**		−**.36**	−**.02**	**.00**	**.001**
Cooperativeness		−.08	−.01	.00	.06
Self-transcendence		.04	.00	.00	.30
Novelty seeking	Depression *R*^2^ = .40	−.01	.00	.00	.71
**Harm avoidance**		**.10**	**.00**	**.00**	**.03**
Reward dependence		−.08	.00	.00	.05
Persistence		−.03	.00	.00	.45
**Self-directedness**		−**.54**	−**.02**	**.00**	**.001**
Cooperativeness		.01	.00	.00	.81
Self-transcendence		.02	.00	.00	.64
Novelty seeking	Anxiety *R*^2^ = .43	.03	.00	.00	.37
**Harm avoidance**		**.46**	**.02**	**.00**	**.001**
Reward dependence		.03	.00	.00	.40
**Persistence**		**.17**	**.01**	**.00**	**.001**
**Self-directedness**		−**.36**	−**.02**	**.00**	**.001**
Cooperativeness		.06	.00	.00	.13
Self-transcendence		.00	.00	.00	.90
**Novelty seeking**	Stress *R*^2^ = .05	**.11**	**.01**	**.01**	**.03**
Harm avoidance		−.01	.00	.00	.82
Reward dependence		.05	.01	.01	.29
**Persistence**		**.14**	**.01**	**.00**	**.001**
**Self-directedness**		−**.11**	−**.01**	**.00**	**.04**
Cooperativeness		.03	.00	.01	.60
Self-transcendence		.05	.00	.00	.26
Novelty seeking	Dysfunction and Suffering *R*^2^ = .02	−.08	.03	.02	.18
Harm avoidance		.01	.00	.02	.91
Reward dependence		−.04	−.02	.02	.39
Persistence		.09	.03	.02	.07
Self-directedness		.04	−.03	.02	.16
Cooperativeness		.09	.04	.02	.08
Self-transcendence		.00	.00	.01	.94

**Notes.**

Significant effects in bold type.

### Osteopathic patients’ personality in comparison to the non-patients

**Figure 7:**
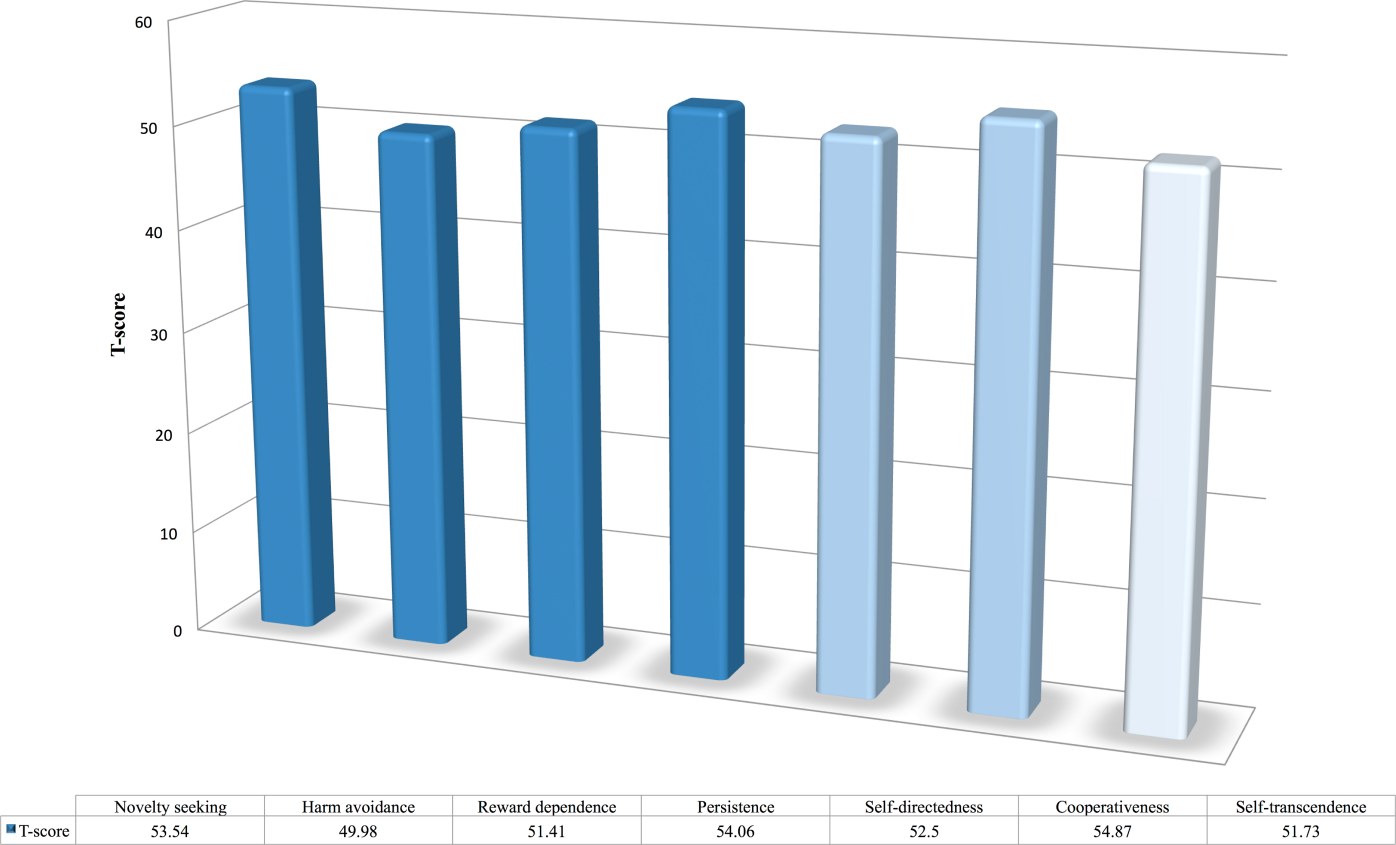
Osteopathic patients’ *T-scores* in the seven personality dimensions. A *T-score* of 50 represents the mean of the population in relation to the control group. 10 points indicate one standard deviation. In other words, deviations of 10 points above or below 50 indicate deviations of one standard deviation from the non-patient group’s mean.

Figure 7 shows the distribution of the *T-scores* of all seven personality dimensions for the osteopathic patients who participated in the present study. The *T-scores* were computed by using the mean and standard deviations of 1,230 individuals from the general Swedish population (S Rosza, pers. comm., 2015). In short, a *T-score* of 50 represents the mean of the control group and 10 points represent one standard deviation. For instance, a *T-score* in cooperativeness of two or more standard deviations below 50 indicates immaturity in the persons’ relations towards others (cf. Cloninger, 2004; Garcia, Anckarsäter & Lundström, 2013). The *T-scores* for the personality dimensions among osteopathic patients ranged between 49.98 and 54.87. The largest divergences among osteopathic patients were found in the temperament dimension of persistence and the character dimension of cooperativeness, but only by about half of standard deviation. The *t-tests*, however, showed that patients did differ in novelty seeking (*t* = 6.94, *df* = 540, *p* < .001, *Cohen’s d* = 0.30), reward dependence (*t* = 2.11, *df* = 540, *p* < .05, *Cohen’s d* = 0.09), persistence (*t* = 7.46, *df* = 540, *p* < .001, *Cohen’s d* = 0.32), self-directedness (*t* = 4.78, *df* = 540, *p* < .001, *Cohen’s d* = 0.21), cooperativeness (*t* = 9.22, *df* = 540, *p* < .001, *Cohen’s d* = 0.40), and self-transcendence (*t* = 2.69, *df* = 540, *p* < .001, *Cohen’s d* = 0.12). Although, these results mean that patients scored higher in six of the seven personality dimensions, the effect sizes were rather small and below the recommended minimum representing a practical significant effect for social science data (see Ferguson, 2009, who recommends a minimum effect size of .41 when using *Cohen’s d* as the effect size estimate).

### Differences in well-being between gender and presenting problem

In the first Multivariate Analysis of Covariance, the independent factors were gender and presenting problem, the dependent factors were the five dimensions of well-being, and age was the covariate variable. Neither gender (*F*(5, 508) = .76; *p* = .58, *Partial η2* = .01, *Wilks’ Lambda* = .99), presenting problem (*F*(10, 1016) = .87; *p* = .56, *Partial η2* = .01, *Wilks’ Lambda* = .98) or the interaction between them (*F*(10, 1016) = .46; *p* = .91, *Partial η2* = .01, *Wilks’ Lambda* = .99) had a significant effect on these five dimensions of well-being. In other words, no differences in well-being were found between males and females, between patients seeking treatment for lower back pain, one problem, or two or more problems. In addition, gender and presenting problem did not interact to influence any of the well-being dimensions.

### Differences in ill-being between gender and presenting problem

In the second Multivariate Analysis of Covariance, the independent factors were again gender and presenting problem, the dependent factors were the five dimensions of ill-being, and age was the covariate variable. Neither gender (*F*(5, 508) = 1.74; *p* = .12, *Partial η2* = .02, *Wilks’ Lambda* = .98), presenting problem (*F*(10, 1016) = .46; *p* = .91, *Partial η2* = .01, *Wilks’ Lambda* = .99) or the interaction between them (*F*(10, 1016) = .70; *p* = .73, *Partial η2* = .01, *Wilks’ Lambda* = .99) had a significant effect on these five dimensions of ill-being. In other words, no differences in ill-being were found between males and females, between patients seeking treatment for lower back pain, one problem, or two or more problems. In addition, gender and presenting problem did not interact to influence any of the ill-being dimensions.

## Discussion

In the present study, we investigated the relationships between personality, well-being and ill-being among Swedish osteopathic patients. The osteopathic patients’ personality was also compared to a healthy control group. Finally, the patients’ health (i.e., well-being and ill-being) was estimated in relation to different presenting problems. This study was intended as a first step to support our proposition of the usefulness of Cloninger’s model as a paradigm for a genuine application of the biopsychosocial model and the persons’ spiritual values in osteopathic practice. Moreover, Cloninger’s personality model is needed to transcend from a patient-centered health care into a person-centered health care by taking in consideration the whole being: body, mind, and soul (i.e., a biopsychosociospiritual approach). Taken together, we found that, as expected, (1) the patients’ personality was related to most of the measures of health (well-being and ill-being) used here, however, the patients’ personality was not related to patients’ self-reported level of dysfunction and suffering in relation to their presenting problem; (2) the osteopathic patients did not differ to a large degree from the control group in any of the personality dimensions; and that (3) patients did not differ in either well-being or ill-being in relation to the problem they seek osteopathic care for.

Personality explained the variance of all well-being measures and four out of five of the ill-being measures (i.e., negative affect, depression, anxiety, and stress). More specifically, self-directedness, which refers to self-determination and being self-sufficient, responsible, reliable, resourceful and effective, was the character trait most positively related to all well-being measures. A person who is mature, responsible, purposeful, relaxed, optimistic, confident and able to control, regulate and adapt behavior in accordance to own goals and values experiences high levels of life satisfaction, harmony in life, positive emotions, energy and is highly resilient as well. Additionally, self-directed individuals experience low levels of negative affect, depression, anxiety and stress. Harm avoidance showed almost the exact opposite pattern by predicting low levels of well-being and high levels of ill-being. That is, a person who is purposeless, blaming, unreliable, ineffective and inert as well as worrying, pessimistic, fearful and fatigable experiences high levels of negative affect, depression and anxiety, which in turn might result in avoidant coping strategies, catastrophizing and misinterpretation of signals (Conrad et al., 2013).

More importantly, self-transcendence was associated positively with harmony in life, resilience, positive affect, and energy, while it was not significantly associated to any of the ill-being measures. This implies that the soul aspect of personality was involved in the patients’ well-being but not in their ill-being. Behaviors, such as acts of kindness, for instance, without a self-transcendent outlook foster cynical distrust and alienation that might end up in ill-being (e.g., cardiovascular disorders, identity problems), rather than well-being (Everson-Rose et al., 2006; Cloninger, Salloum & Mezzich, 2012). The role of self-transcendence as a crucial ingredient in any behavior, or in this case interventions, in fostering well-being is a central issue for osteopathic practice as its philosophy sees the person as a unity of being (i.e., body, mind, and soul) (e.g., Penney, 2013). For example, when the patient and the osteopath agreed upon specific interventions, treatment, or changes in lifestyle, it is crucial to engage the spiritual values of the person if the treatment is going to lead to, besides alleviation of pain, also well-being. If the patient only believes (i.e., mind) what the practitioner tells her/him, but does not see a meaning or has deep trust (i.e., soul) in the treatment, then she/he might follow the given advices (i.e., body) without having the benefits of long-lasting well-being by integrating the activities to her/his daily life and thereby increase her/his ability to be resilient if the problem returns (cf. Hunter, 2007). In this context, without including a reliable measure of the person’s spiritual values, osteopaths may lack the genuine knowledge of the person’s ability to overcome adversity and to return to her/his previously established functional baseline (i.e., resilience), as well as the ability to have a sense of meaning in life that gives a sense of balance in life (i.e., harmony), and allows the individual to experience positive emotions (i.e., positive affect and energy).

Regarding positive emotions, individuals who experience high levels of this type of emotions experience lower levels of pain (Sturgeon & Zautra, 2010; Sturgeon & Zautra, 2013). Self-transcendence, on the other hand, permits the individual to accept failures and tolerate uncertainty and ambiguities as well as identify the self with a “bigger whole” (Cloninger, 2004), which probably enables her/him to adapt well in circumstances involving pain and losses (Sturgeon & Zautra, 2010; Sturgeon & Zautra, 2013). Indeed, resilience involves positive coping strategies, such as, positive reinterpretation and growth (Smith & Zautra, 2008), which may help individuals with pain. For example, while a person’s tendency to avoidant approach behavior (i.e., high harm avoidance) makes her/him more susceptible to negative pain-related outcomes, high levels of self-transcendence might help her/him to experience momentary thoughts of pain acceptance that makes her/him more resilient (cf. Sturgeon & Zautra, 2013). For the osteopathic practitioner, increases in pain acceptance after/under manual treatment interventions done over time, might indicate that the patient has grown in self-awareness or self-transcendence, which in turn might help the patient even during times of increased pain or stress (cf. Sturgeon & Zautra, 2013), rather than only flourishing under convenient external conditions. In other words, this covers a complete notion of good health including resilience and flourishing in both negative and positive conditions, respectively (Cloninger, Salloum & Mezzich, 2012).

Although these findings are in concordance with the literature (e.g., Knaster et al., 2012; Celikel et al., 2009; Cloninger & Zohar, 2011; Eley et al., 2013), none of the personality traits were associated to the patients’ self-reported level of pain-related dysfunction and suffering. Earlier research has showed that high levels of harm avoidance and low levels of self-directedness are linked to chronic pain (Conrad et al., 2013; Conrad et al., 2007; Malmgren-Olsson & Bergdahl, 2006). Researchers even suggest that personality traits can be viewed as predisposing factors for developing chronic pain (Conrad et al., 2013). Nevertheless, most research has been conducted among chronic pain patients (see for example Dersh, Polatin & Gatchel, 2002; Keefe et al., 2004; Linton & Shaw, 2011) or patients with psychiatric disorders (Conrad et al., 2013), who exist within a very different context (i.e., belonging to a medical department) than the osteopathic patients in the present study (i.e., outpatient care). Thus, explaining the non-significant relationship between personality dimensions and pain-related dysfunction and suffering in the present study. That being said, it is reasonable to question the non-significant relationships between personality and self-reported pain-related dysfunction and suffering in the present study. We suggest that these non-significant findings may indicate some measurement problems with regard to the dysfunction and suffering variable used here—we measured discomfort associated to the pain, rather than the pain being acute or chronic. Moreover, there are other measurements of pain that are more suitable for clinical practice that include a multifaceted notion of pain, such as the perspective of time and whether the presenting problem is acute or chronic in nature (e.g., the Brief Pain Inventory; Cleeland, 1989). Other measures of pain even specify whether or not the discomfort was specific to work-life, family-life, general activity, mood, walking, relations with others, sleep, and enjoyment of life. Such measures accommodate biological, psychological, and social aspects of pain-related dysfunction and suffering (cf. Cloninger, Salloum & Mezzich, 2012) better than the one used here.

Osteopathic patients did not differ considerably from the control group in the seven personality dimensions. Nevertheless, two of the bigger differences were found in the temperament dimension of persistence and the character dimension of cooperativeness. Individuals with high levels of persistence are hardworking, do not give up easily and, persevere through hard and difficult moments (Cloninger, 2004). This observation may suggest that individuals seeking osteopathic care drive themselves through difficulties that cause pain in different parts of the body. Moreover, as osteopathic care is not covered by social insurance in Sweden, those who end up in an osteopathic clinic may as well be patients that have been visiting other regulated health care providers without experiencing proper relief of their problem thereby mirroring their tendency to persevere through hardship. However, as for the rest of the temperament dimensions, there are no good and bad temperaments (Wong & Cloninger, 2010). Indeed, in the present study, the patients’ score in persistence was one of the best predictors of high levels of resilience and positive affect, but also one of the predictors of high levels of anxiety and stress. In contrast, the patients’ scores in the three character traits were only positively related to well-being and only negatively related to ill-being. Thus, a highly persistent individual may, with increased character development, be a healthy and resilient patient. With respect to cooperativeness, it is difficult to speculate in a plausible reason to why osteopathic patients scored higher in this character trait compared to the controls. Nevertheless, cooperativeness is an indicator of willingness to stop destructive behaviors, such as smoking (e.g., Bishry et al., 2012). Thus, if it is the case that osteopathic patients are more cooperative than the general population, osteopaths may be able to utilize these type of values and goals more easily with their patients so that they can make self-directed choices based on cooperative values to terminate unhealthy behaviors.

In addition, we did not found differences in either well-being or ill-being between osteopathic patients as a function of body pain location. Together with earlier findings suggesting a personality-chronic pain relationship (e.g., Conrad et al., 2013), this implies that the effects of the pain, rather than the actual site of the pain, is what determine how it affects the patients’ health. The lack of differences in health between osteopathic patients with different presenting problems also implies that, regardless of where the pain is located, osteopaths can and ought to focus on different aspects of the person’s health. Nevertheless, it may be argued that the division of the sample into three presenting problem groups is inappropriate. Nevertheless, comparisons using all six presenting problem groups were also conducted as a preliminary part of the study and no differences were found between groups. We chose to group patients into three larger groups, especially since low back pain remains the most common pain condition across the world (Hoy et al., 2012; Manchikanti et al., 2009). Low back pain is also highly represented in research relating to chronic pain and psychological variables (see for example Linton, 2000; Dersh, Polatin & Gatchel, 2002) and personality (Conrad et al., 2007).

### Limitations and further suggestions

Besides those already stated, another limitation was the response rate and the unbalanced gender distribution. Out of 5,198 known possible patients only 545 completed the survey (see Fig. 2). The osteopaths were the ones who emailed the largest amount of patients, thus, we were not able to control for reminders or whether or not the patients actually opened the emails with the link to the survey. In contrast, out of the 343 emails send by the research team, 189 emails were opened and 154 were never opened. Nevertheless, the final number of valid responses (*N* = 524) is perhaps not optimal, but this level of attrition rate is common in web-based as opposed to paper-based surveys (Shih & Fan, 2008). That being said, the attrition rate undermines the generalizability of the present results. Moreover, the responses are also at risk for bias as the individual capacity to use and access technology may vary (Atif, Richards & Bilgin, 2012). The control group may not be representative of the Swedish population; consequently, it is still uncertain if the osteopathic patients actually differ in personality compared to Swedish non-patients.

The large sample size may have produced small standard errors, which is more likely to produce statistically significant results. In the present study, even co-variances among residuals/errors were significant. Although these co-variances among errors were added in order to improve the fit of the model, it might also indicate some of the limitations and usefulness of linear models to investigate non-linear dynamic adaptive systems, such as, personality (cf. Cloninger, Svrakic & Svrakic, 1997). Instead of using linear or population-oriented methods, future studies may benefit from using person-oriented methods detailed in recent literature (see among others Bergman & Lundh, 2015; Cloninger, Svrakic & Svrakic, 1997; Lundh, 2015; Von Eye & Wiedermann, 2015; Vargha, Torma & Bergman, 2015; Garcia, MacDonald & Archer, 2015; Wong & Cloninger, 2010).

Another limitation is the fact that sociodemographic factors were not controlled for. After all, sociodemographic factors, as well as sociocultural and socioeconomic factors, interact and influence the scope of pain and physical pathology (Gatchel et al., 2007; McBeth & Jones, 2007; Henschke, Kamper & Maher, 2015). Nevertheless, we opted for only using personality as the main variable of interest because in the context of well-being in western societies, personality factors overrule the impact of sociodemographic factors (Lucas & Diener, 2008; see also Mokdad et al., 2004). Additionally, in Sweden, osteopathic care is not covered by social insurance; thus, most of those who seek osteopathic care are probably a relatively homogeneous group when it comes to socioeconomic variables. Indeed, almost 81% of the participants here reported being employed for wages (see Table 2). Finally, although the cross-sectional design fitted the aim of the study, it cannot imply causality. Future studies ought to address the effects of specific osteopathic treatment techniques on character development and the relationship between character-pain-health through, for example, randomized controlled trials.

### Implications and concluding remarks

The osteopathic philosophy and approach underpins a health-orientated and holistic view of the human being where the body, mind, and soul are interdependent in both health and disease. Nevertheless, osteopathic practice has been questioned both in how it is distinguished from other healthcare practices with regard to the biopsychosocial model (Tyreman, 2013) and as to how the claimed patient-centeredness and spiritual aspects are incorporated into clinical practice (Thomson, Petty & Moore, 2013). Acknowledging the need of a person-centered approach in which the patients’ personality can be measured using a biopsychosociospiritual model with a long and robust scientific history creates a completely different outlook for osteopathy. The first finding, for instance, suggested that the patient’s personality as a ternary construct comprising body, mind, and soul, is associated to both well-being and ill-being. Thus, Cloninger’s biopsychosocial model of personality is a valid paradigm applicable to osteopathic philosophy and practice. The lack of substantial differences in personality between patients and controls implies that osteopaths might, with proper education, coach their patients into greater self-awareness. In other words, osteopathic patients in general do not seem to suffer of mental disorders and therefore their character development might not require psychiatric interventions. In this context, an osteopath who has proper training (see http://anthropedia.org for well-being coaching programs) on how to understand personality profiles has the advantage to recognize a patient with mental health problems or with risk for developing a disorder and then re-direct the patient to proper care. Finally, the lack of differences in both well-being and ill-being between osteopathic patients with different presenting problems implies that osteopaths should focus on different aspects of the patients’ health regardless of where the pain is located.

A genuine and intuitive knowledge about the patient’s personality may facilitate the practitioner’s planning and delivery of treatment, and also improve and promote empathetic communication and advises in consultations (Wong & Cloninger, 2010). We argue that knowledge about the patients’ personality will guide the practitioner to recognize stress reactions, different coping strategies for prevention and early treatment or handling of musculoskeletal disorders (cf. Ahlberg et al., 2002). In addition, this systematic knowledge about the patients’ personality, if used to help the patient in her/his own path to self-awareness, will promote health and behaviors that empower the individual to take care of her/his body and whole being. Importantly, just telling people what they should do to reduce stress and modify their lifestyle choices is ineffective unless the whole person can be helped to become aware of the causes of their condition and motivated to make changes (Cloninger, Salloum & Mezzich, 2012).

As a first phase in this process of mutual self-awareness, the practitioner would benefit from awareness of her/his own being in order to understand unconscious motives and drives that may lead to conflicts within her/himself and between her/himself and the patient. As a second phase, the patient needs to become aware of her/his own temperament and character combination. If the practitioner is able to communicate in a non-judgmental manner to the patient about the patient’s own personality it might help the patient to become aware of behaviors that generate ill-being, including those behaviors that cause any presenting problem (cf. Wong & Cloninger, 2010). Additionally, such non-judgmental communication might also promote the patient’s ability to make self-directed choices that foster well-being (cf. Cloninger, 2004; Wong & Cloninger, 2010). From this point of view, the patient-centered approach may then evolve to a person-centered approach, which indeed fits better with the nature of osteopathic philosophy. For example, patient-centered care generally concentrates around the management of diseases and generally refers to interactions in visits, view the body systems as distinct, and may be episode-oriented; whilst person-centered care refers to interrelationships over time, views diseases and body systems as interrelated phenomena, and considers episodes as part of life-course experiences with health (for a detailed description of the distinction between patient-centered and person-centered approaches see Starfield, 2011).

True and lasting cultivation of well-being develops as a result of self-awareness (Cloninger, 2008). To help a patient to become aware of different behaviors does not necessarily imply that psychological intervention should be implemented in the osteopathic practice. For instance, simple techniques in communication (Engel, 1980; Linton & Shaw, 2011) and very subtle changes in clinical practice (Eccleston, 2001) have significant effects in the patient’s health. Nevertheless, these subtle changes in practice do probably involve large changes within the practitioner her/himself, such as, the practice of forethought to cultivate intuitive wisdom (cf. Cloninger, Salloum & Mezzich, 2012). In recent years, for example, well-being coaching training modules have been developed specially for health care practitioners to coach patients in their development of self-awareness (Cloninger & Cloninger, 2011a; Cloninger & Cloninger, 2011b). Finally, another type of modus operandi is to give care and treatment from several specialties (e.g., osteopaths and well-being coaches), which is also more in line with the view of multi-morbidity in the person-centered approach to health.

*“I...a universe of atoms, an atom in the universe.”*

Richard P. Feyman

*“Think often of the bond that unites all things in the universe and their dependence upon one another. All are, as it were, interwoven and in consequence linked in mutual affection”*

Marcus Aurelius

## Additional Information and Declarations

### Data Availability

The following information was supplied regarding data availability: The raw data is available upon request to the Network for Empowerment and Well-Being, lead researcher Danilo Garcia: http://celam.gu.se/people/danilo-garcia.
